# Increasing the Efficiency of CRISPR/Cas9-mediated Precise Genome Editing of HSV-1 Virus in Human Cells

**DOI:** 10.1038/srep34531

**Published:** 2016-10-07

**Authors:** Chaolong Lin, Huanhuan Li, Mengru Hao, Dan Xiong, Yong Luo, Chenghao Huang, Quan Yuan, Jun Zhang, Ningshao Xia

**Affiliations:** 1 State Key Laboratory of Molecular Vaccinology and Molecular Diagnostics, National Institute of Diagnostics and Vaccine Development in Infectious Diseases, School of Public Health, Xiamen University, Xiamen, 361102, China; 2 School of Life Sciences, Xiamen University, Xiamen, 361102, China

## Abstract

Genetically modified HSV-1 viruses serve as promising vectors for tumour therapy and vaccine development. The CRISPR/Cas9 system is one of the most powerful tools for precise gene editing of the genomes of organisms. However, whether the CRISPR/Cas9 system can precisely and efficiently make gene replacements in the genome of HSV-1 remains essentially unknown. Here, we reported CRISPR/Cas9-mediated editing of the HSV-1 genome in human cells, including the knockout and replacement of large genes. In established cells stably expressing CRISPR/Cas9, gRNA in coordination with Cas9 could direct a precise cleavage within a pre-defined target region, and foreign genes were successfully used to replace the target gene seamlessly by HDR-mediated gene replacement. Introducing the NHEJ inhibitor SCR7 to the CRISPR/Cas9 system greatly facilitated HDR-mediated gene replacement in the HSV-1 genome. We provided the first genetic evidence that two copies of the ICP0 gene in different locations on the same HSV-1 genome could be simultaneously modified with high efficiency and with no off-target modifications. We also developed a revolutionized isolation platform for desired recombinant viruses using single-cell sorting. Together, our work provides a significantly improved method for targeted editing of DNA viruses, which will facilitate the development of anti-cancer oncolytic viruses and vaccines.

Herpes simplex virus type 1 (HSV-1) is a highly epidemic pathogen, which infects approximately 60% of the population worldwide^1^. HSV-1 is mainly transmitted through oral-oral contact and causes orolabial herpes. A noteworthy outcome of HSV-1 infection is the rare but fatal occurrence of neonatal herpes in pregnant women^2^. HSV-1 displays a set of notable features that make it a suitable viral vector for therapeutic purposes. HSV-1 harbours a large genome of 152 kb, containing approximately 77 genes, half of which are not essential for virus replication^3^. Various locations and genes within the HSV-1 genome have been identified as editable, without these genes, the virus can progress through its entire life cycle only in cancer cells, but not in normal cells. Modified HSV-1 recombinants carrying different therapeutic agents or with the removal of some unessential genes have demonstrated with good efficacy in several clinical trials aimed at treating cancers^4^,^5^,^6^.

Manipulation of viral genomes is a powerful strategy for studying viral gene function and constructing attenuated viral vaccine and gene therapy vectors. Various genome-editing methods, such as BAC recombination and homolog recombination, have been employed to modify the HSV-1 genome *in vitro*^7^,^8^. These methods are generally based on homologous recombination to insert a fragment of DNA containing a drug selectable marker into a target gene or to replace a gene with a visualized gene^9^. However, these strategies are time-consuming and inefficient, as they require several rounds of selection and transfer vector cloning, as well as needing large scale of screening processes^10^. Additionally, the site of DNA insertion or replacement when using these methods is often random, which can result in some unwanted insertions and deletions at nearby homologous regions^3^.

Recently, more efficient and site-specific genome editing technologies, such as zinc finger nuclease (ZFN) and transcription activator-like effector nuclease (TALEN)-mediated gene editing have been developed to target any gene of interest within any organism^11^,^12^. However, these methods have not been successfully adapted for editing genes in DNA viruses carrying large viral genomes. The CRISPR/Cas9 (Clustered regularly interspaced short palindromic repeats/CRISPR-associated 9) system has substantially advanced efforts in specific gene editing and has been successfully applied to modify the episomal genomes of human and other organisms^13^,^14^,^15^. The CRISPR/Cas9 system utilizes a prokaryotic RNA-guided programmable nuclease that can make a double-strand DNA break (DSB) at a specific site under the guidance of a leading RNA. This DSB process depends on the co-expression of two basic components: a guide RNA (gRNA) and Cas9 nuclease. Making a specific DSB can trigger DNA repair via either error-prone non-homologous end joining (NHEJ) or homology-directed repair (HDR). In the presence of the CRISPR/Cas9 system, the NHEJ inhibitor SCR7 was proven to increase the efficiency of Cas9-mediated HDR by at least by 7-fold in mammalian cells^16^. Although CRISPR/Cas9 system has been introduced comprehensively since 2014, this technique normally is adapted to directly edit the genomes of organisms, which can reproduce independently. However, viruses depend on the host cells that they infect to reproduce, so the genome editing procedure of them is more complicated and difficult than other self-reproducing organisms. It’s worth to investigate the applicability and feasibility of CRISPR/Cas9 system in editing viruses. The CRISPR/Cas9 technique has been reported to increase the gene knock-in frequency in adenovirus and HSV-1 genomes using traditional transfection/infection methods in 293T cells^17^,^18^. However, whether CRISPR/Cas9 method can precisely and efficiently edit genes or make gene replacements in the HSV genome remains essentially unknown. NHEJ-directed repair has been efficiently induced during CRISPR/Cas9-mediated genome editing, which may restrict the frequency of HDR. However, it remains to be determined whether CRISPR/Cas9 can further increase the efficiency of HDR for gene replacement on the HSV-1 genome by suppressing the NHEJ pathway. In addition, it is of interest to know whether the two-copy genes on a single HSV-1 genome might be susceptible to CRISPR/Cas9-mediated editing. One major concern regarding CRISPR/Cas9-mediated genome editing is the specificity of gRNA, as a lack of specificity can potentially cause off-target modifications in the targeted genomes^14^,^19^. To avoid such unspecific targeting, methods are needed to minimize possible off-target sites in the HSV-1 genome.

In this study, we evaluated the feasibility and specificity of using CRISPR/Cas9-mediated gene replacement to modify the HSV-1 genome in human cells stably expressing CRISPR/Cas9. We reported an efficient method based on CRISPR/Cas9 technology to precisely edit the HSV-1 genome, including the ability to perform gene knockout and gene replacement. We also established a program for specific gRNA selection to minimize potential off-target effects on the HSV-1 genome. Large fragments of the HSV-1 genome could be replaced with any other type of genes with significantly improved efficiency. Introducing the NHEJ inhibitor SCR7 to the CRISPR/Cas9 system greatly facilitated HDR-mediated gene replacement on the HSV-1 genome in human cells. We also reported a rapid, revolutionized method using single-cell sorting, which will significantly improve the isolation efficiency of desired recombinant viruses. We provided the first genetic evidence that two copies of the same gene in different locations on the HSV-1 genome could be simultaneously modified with improved efficiency. Our aim was to precisely and efficiently construct a recombinant HSV-1 virus that does not contain any unwanted mutations outside the targets. This method could serve as a foundation for editing other DNA viruses carrying large genomes and will potentially be useful to further optimize HSV-1-based oncolytic vectors for cancer therapy and genetically attenuated vaccines for infection control.

## Results

### CRISPR/Cas9-mediated indels at specific sites on the HSV-1 genome

**Figure 1:**
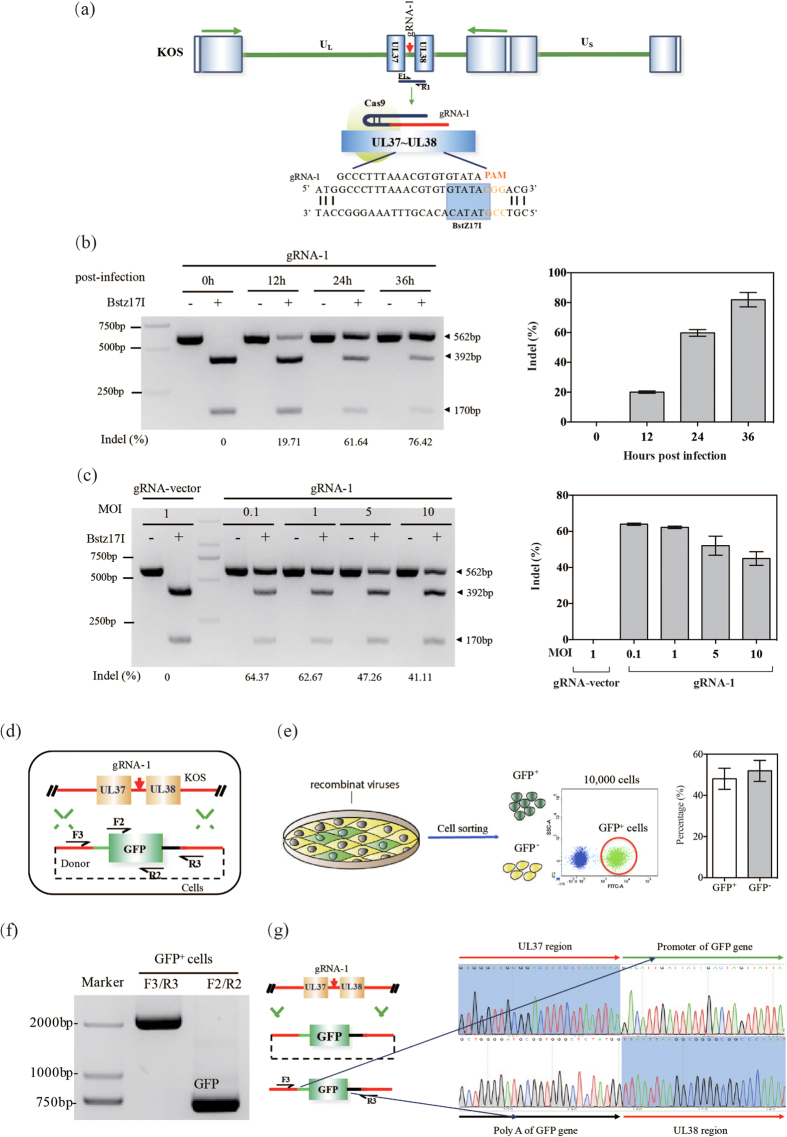
CRISPR/Cas9-mediated editing of the HSV-1 genome in cultured human cells. (**a**) Schematic diagram of the HSV-1 genome and the gRNA-1 target site nearby the BstZ17I enzymatic site. The PAM motif is highlighted in orange. (**b**) Fragments amplified from cells harvested at different time points were used for BstZ17I enzymatic analysis. (**c**) Fragments amplified from the cells infected with different MOIs were used for BstZ17I enzymatic analysis. (**d**) Strategy for GFP gene knocking-in the UL37/UL38 region of the HSV-1 genome. (**e**) Strategy for isolation of GFP^+^ viruses using single-cell sorting. (**f**) Verification of HSV-GFP recombinants by PCR analysis. (**g**) Sequence analysis of the upstream and downstream region of the knock-in.

First, we chose the commonly used HSV-1 KOS strain to study whether the HSV-1 genome could be efficiently cleaved using the CRISPR/Cas9 system. To exclude the effects of viral gene modification on viral replication, we selected a gRNA-1 to target a specific non-coding site in the region between UL37 and UL38, which theoretically had no effect on viral function (Fig. 1a). The PAM sequence of the gRNA-1 was located right after the BstZ17I enzymatic site, such the CRISPR/Cas9-induced cleavage would destroy this BstZ17I enzymatic site. After 24 hour’s transfection, 293T cells were infected with KOS at different multiplicities of infections (MOIs), and the cells were harvested when cytopathic effects were observed. The efficiency of the CRISPR/Cas9-mediated cleavage was highest at a MOI of between 0.1 and 1, and it decreased at a higher MOIs (5 or 10), likely due to the presence of excess uninfected virus. We next investigated the effect of viral infection time on the efficiency of CRISPR/Cas9-mediated cleavage. The cleavage efficiency increased with increasing viral infection time and reached a peak at 36 hours’ post-infection (Fig. 1b). We then investigated the effect of viral infectious dose on the efficiency of CRISPR/Cas9-mediated cleavage. The cleavage efficiency increased with increasing viral infectious dose and reached a peak at a MOI of 1 PFU/cell (Fig. 1c). Next we investigated whether foreign genes could be feasibly knocked in and effectively isolated. To accomplish this, we employed the Cas9/gRNA-1 system and a repair donor (pTUL37/38GFP) containing a GFP reporter flanked by 710-bp of the 3′ untranslated region of UL37 and 710-bp of the 5′ untranslated region of UL38 to introduce the GFP gene into the cleavage site by HDR-mediated recombination (Fig. 1d). The 293T cells were re-infected with the Cas9-edited recombinant viruses and subjected to cell sorting by flow cytometry. GFP^+^ cells containing HSV-GFP viruses were isolated at a percentage of approximately 48% (Fig. 1e) and verified by PCR analysis (Fig. 1f). The results showed that the GFP gene was successfully knocked in at the junction of the UL37 and UL38 regions (Fig. 1g). Collectively, these results show that the CRISPR/Cas9-mediated cleavage was efficient and could also be repaired via HDR.

### Efficient cleavage and functional examination of the HSV-1 genome in cells expressing CRISPR/Cas9

**Figure 2:**
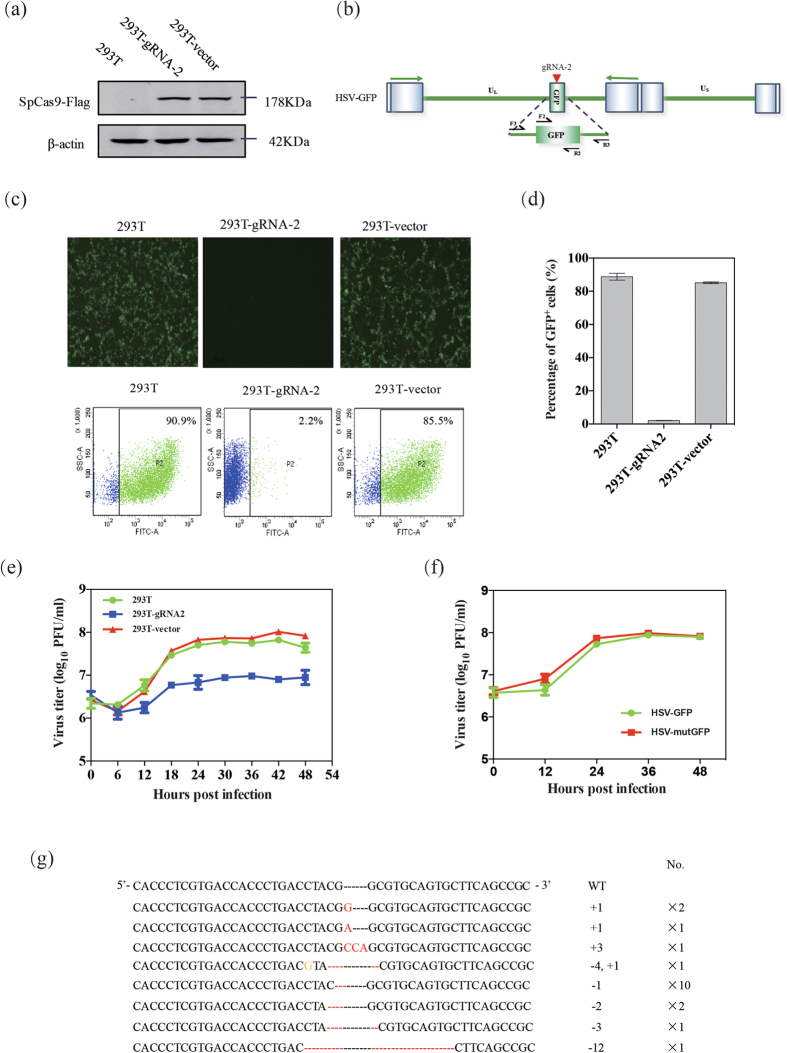
Editing of the HSV-1 genome in cells stably expressing CRISPR/Cas9. (**a**) Verification of stable Cas9 expression. (**b**) Schematic of the EGFP gene in the HSV-1 genome with the targets for gRNA-2 indicated by the red arrowhead. (**c**) Efficient cleavage of the GFP gene in the HSV-1 genome. (**d**) The percentage of the GFP^+^ cells analyzed by flow cytometry. (**e**) Titration of HSV-GFP recombinants after CRISPR/Cas9 editing at different harvest time points. (**f**) Titration of HSV-mutGFP and HSV-GFP viruses in U-2 OS cells after infection at a MOI of 0.01. **(g**) Sequences of indels in the GFP region of the HSV-1 genome were identified and shown.

**Figure 3:**
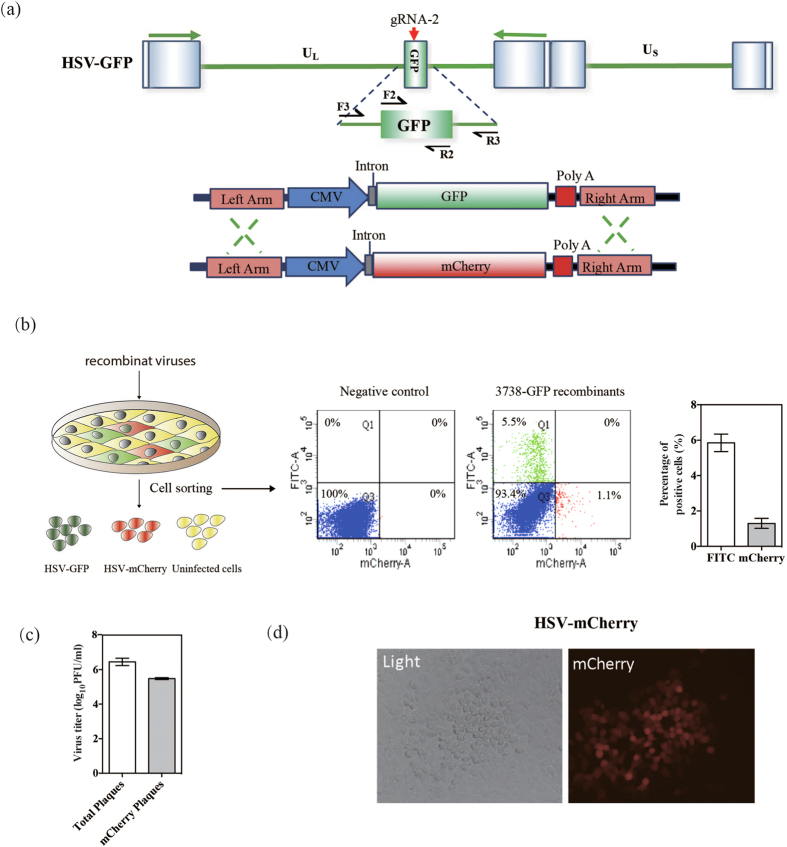
Feasibility of CRISPR/Cas9-mediated Gene replacement in the HSV-1 genome. (**a**) Schematic and strategy for Cas9/gRNA-2-mediated HDR for replacing GFP with mCherry. (**b**) Strategy for isolation of mCherry^+^ and/or GFP^+^ viruses using single-cell sorting. (**c**) Titration of recombinant viruses was performed. (**d)** Images of plaques with red fluorescence are shown.

Next, we expanded our study to examine whether the high-efficiency genome cleavage discussed above could be used to study the functions of target genes. To accomplish this, we selected a gene encoding enhanced green fluorescent protein (EGFP) carried by a recombinant HSV-GFP as a target gene. gRNA-2 was designed to target the coding sequence of the EGFP gene (Fig. 2a). 293T-Cas9/gRNA cells constitutively expressing the Cas9 and the corresponding gRNA were established and chosen as the host cells for CRISPR/Cas9-mediated editing. In these cells, the expression of Cas9 protein was detected by western blotting, and the stably expressed Cas9/gRNA-2 likely resulted in the maximal cleavage of the infected viruses (Fig. 2b). After 24 hours’ infection, the presence of Cas9/gRNA-2 efficiently abolished the fluorescence in HSV-GFP-infected cells, which indicated that the GFP gene was mutated following CRISPR/Cas9-mediated cleavage (Fig. 2c,d). The viral yields of HSV-GFP viruses in the 293T-Cas9/gRNA-2 cells at different time points were all lower than those in the control cells, which suggested that CRISPR/Cas9-mediated cleavage and repair might affect viral production (Fig. 2e). The results demonstrated that the cleavage efficiency reached a relatively high level at 24 hours’ post-infection and the viruses were edited and produced within a single replication cycle. Interestingly, a substantial amount of virus was recovered following site-specific cleavage via NHEJ. We next examined whether mutated parental viruses could be inheritably passed down to progeny viruses by infecting 293T cells with the parental viruses and quantifying the resultant fluorescence intensity via microscope. Fluorescence was rarely observed in these cells (Fig. S4), which suggested that the genomes of the progeny viruses were effectively inherited from the mutated parental viruses. Next, we separated the HSV-mutGFP viruses (GFP^−^) from the mixed pool of edited HSV-GFP viruses, the viral yields of HSV-mutGFP viruses in U-2 OS cells were similar with those of HSV-GFP (Fig. 2f and Fig. S5), which revealed that the mutations on the GFP gene of HSV-GFP genome might not affect the viral replication. Additionally, the diversity of the mutations at the cleavage site on the GFP gene was analyzed by sequencing the PCR fragments amplified from the mutated parental viruses. The PCR products were cloned into T vectors for direct sequencing. Among the 19 clones selected, all clones had indels nearby the cleavage site, 4 clones had nucleotide insertions, and 15 clones had nucleotide deletions — 10 of these 15 clones had a missing G (Fig. 2g). Missing nucleotides were frequently observed and thought to be dominant mutations. Given that the GFP gene was a functional but unessential gene in the HSV-1 genome, our results indicated that viral genomes could be functionally edited and repaired by cellular repair systems. Moreover, we employed a mCherry reporter to test the feasibility and efficiency of gene replacement of GFP reporter in the HSV-GFP genome, which the signal of parental viruses could be distinguished (Fig. 3a). It showed that GFP^+^ cells maintained a 5:1 ratio with mCherry^+^ cells when we assayed the recombinants by reinfection coupled to single cell-sorting (Fig. 3b). The efficiency of gene replacement of GFP reporter with mCherry reporter in the genome of HSV-GFP was approximately 10.2% (Fig. 3c), and we then successfully obtained mCherry^+^ viruses (Fig. 3d).

### The replacement of genes on the HSV-1 genome by CRISPR/Cas9-mediated HDR

**Figure 4:**
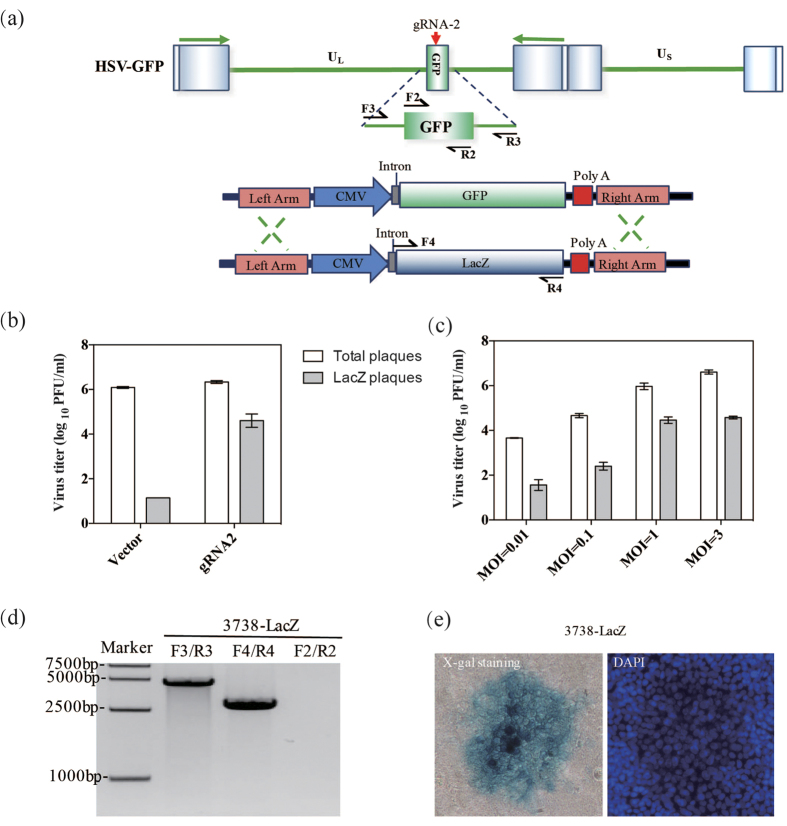
Gene replacement in the HSV-1 genome by CRISPR/Cas9-mediated HDR. (**a**) Schematic and strategy for Cas9/gRNA-2-mediated HDR for replacing GFP with LacZ. (**b**) Titration of recombinant viruses was performed. (**c**) The efficiencies of HDR-mediated gene replacement at different MOIs. (**d**) Genomic DNA extracted from 3738-LacZ was purified and subjected to PCR analysis. (**e**) Images of blue plaques and DAPI-stained nuclei are shown.

Because of the applicability of HSV-1 vectors for gene therapy, multiple viral genes were replaced with different therapeutic genes. First, the efficiency of HDR-mediated gene replacement on the HSV-1 genome in Cas9/gRNA-expressing cells was examined, and then factors affecting the efficiency of gene replacement by the CRISPR/Cas9 system were further evaluated. To accomplish these goals, we constructed a repair donor (pTUL37/38lacZ) containing a LacZ reporter, which was an indicator with large DNA size (Fig. 4a). To prevent the cleavage of the repair donor by the Cas9/gRNA complex, we chose the gRNA-2 targeting the GFP coding region as the guiding RNA. Using this system, we expected to obtain LacZ^+^ viruses that could form blue plaques after staining with X-gal substrates. The results showed that the efficiency of the naturally occurring HDR was lower than 10^−5^; however, the efficiency of CRISPR/Cas9-mediated HDR of large gene was significantly increased to approximately 2.1%, which has never been reported before (Fig. 4b). As we could see, the efficiency of HSV-LacZ recombination was lower than that of HSV-mCherry recombination. We next explored the effect of virus quantity on the efficiency of HDR-mediated gene replacement in the above-mentioned CRISPR/Cas9 cells. The gene replacement efficiency increased with increasing MOI and reached a peak at a MOI of 1 PFU/cell (Fig. 4c). As expected, higher viral yields were obtained as the MOI increased (0.01–3), but the recombination efficiency only increased up to a MOI of less than 1 PFU/cell due to the presence of excessive virus at higher MOIs. We PCR-amplified several regions of the viral genome, including the GFP gene, the LacZ gene and the region flanking the UL37/UL38 genes. All amplified fragments were shown to possess the expected size (Fig. 4d). Particularly, the intact replacement of a sizable genomic fragment with a target gene was verified by sequencing the amplification product using primers targeting the flanking regions of the target gene. An individual blue plaque representing 3738-LacZ virus was observed (Fig. 4e).

### Introduction of the NHEJ inhibitor SCR7 during CRISPR/Cas9-mediated editing of the HSV-1 genome

**Figure 5:**
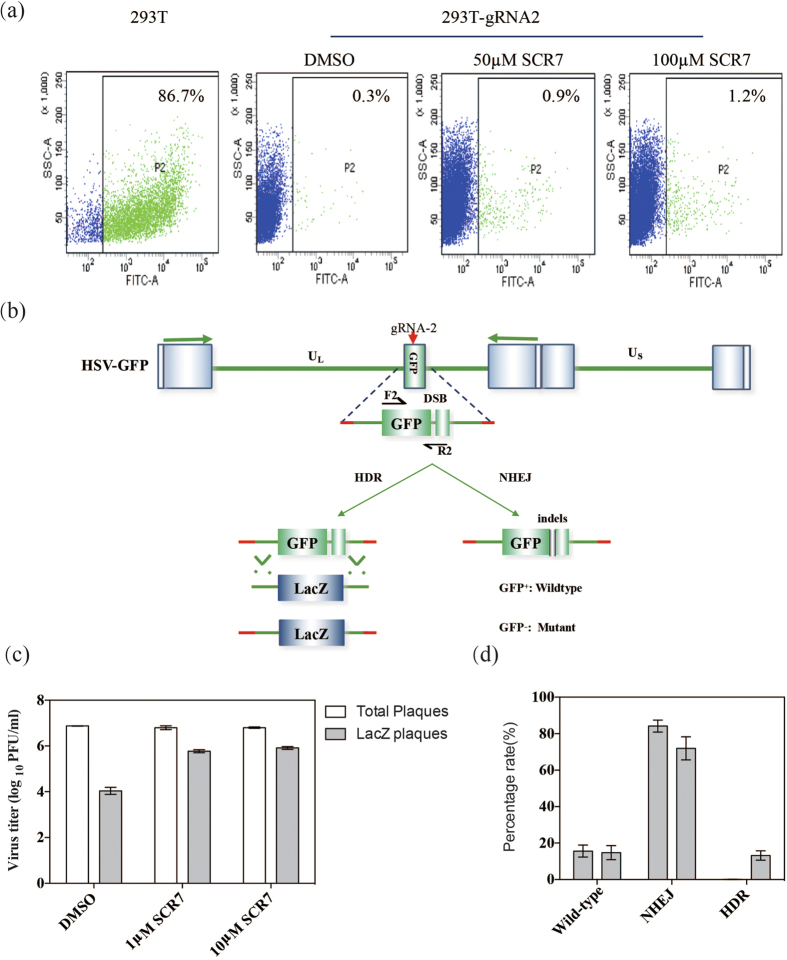
Improving the HDR-mediated recombination efficiency for the HSV-1 genome by introducing the NHEJ inhibitor SCR-7. (**a**) Recovery of GFP^+^ recombinant viruses from 293T-gRNA-2 cells after the addition of the indicated concentrations of SCR7. (**b**) Schematic diagram of HDR- and NHEJ-directed repair after CRIPSR/Cas9-mediated cleavage of the GFP gene in HSV-GFP. (**c**) HDR-mediated recombination efficiency in the presence of SCR-7. (**d**) Viral titers representing wild-type, NHEJ and HDR are shown.

The previous data showed that a number of our Cas9-edited HSV-1 viruses were not GFP-positive, indicating that a substantial number of the viruses had been repaired by NHEJ rather than by HDR with the donor plasmids. Therefore, it was questioned whether introducing an NHEJ inhibitor could further improve the recombination efficiency of a desired recombinant virus. To answer this question, 293T-gRNA2 cells were pre-incubated with different concentrations of the NHEJ inhibitor SCR7 (0~100 μM) and infected with HSV-GFP at a MOI of 1 PFU/cell. The cells were harvested two days’ post infection and subjected to flow cytometry analysis. Interestingly, the percentage of GFP^+^ cells produced by intra-molecular self-rescue slightly increased as the concentration of SCR7 increased, likely due to the partially inhibited NHEJ pathway (Fig. 5a). It was noted that the recovery efficiency of HSV-GFP was very low when the donor plasmids were absent. Next, we tested the HDR-mediated recombination efficiency with the help of SCR7 and the donor plasmids. The LacZ gene was expected to replace the GFP gene in the HSV-GFP genome in this system (Fig. 5b). As expected, the recombination efficiency of CRISPR/Cas9-mediated HDR was significantly increased by >10-fold when the 1 μM of SCR7 was introduced; however, the higher concentration of SCR7 did not show further benefits in improving the replacement efficiency (Fig. 5c). Moreover, the percentage of NHEJ-directed repair was reduced when the SCR7 was added, which was calculated by the number of GFP^−^LacZ^−^ viruses in the total population of recombinant viruses. Meanwhile, the percentage of HDR-mediated repair was significantly increased when examining the number of LacZ^+^ viruses. Introducing SCR7 into the CRISPR/Cas9 system could greatly improve the gene replacement efficiency on the HSV-1 genome by inhibiting NHEJ-directed repair, which would facilitate the production of desired recombinant viruses (Fig. 5d).

### Synchronously replacing two copies of the same gene on the HSV-1 genome by CRIPSR/Cas9-mediated gene editing

**Figure 6:**
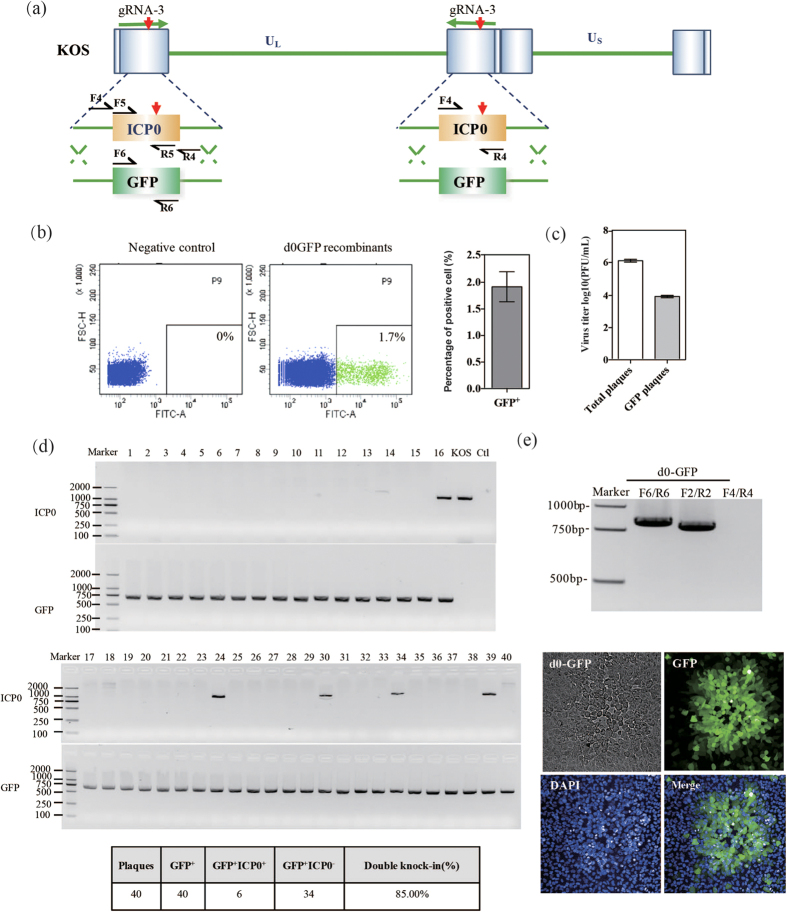
Synchronously replacing two copies of the same gene on a single HSV-1 genome. (**a**) Schematic and strategy for replacing ICP0 with GFP. (**b**) Flow cytometry analysis of the HSV-GFP recombinants by reinfection coupled to single-cell sorting. (**c**) Titration of HSV-GFP recombinants after CRISPR/Cas9 editing. (**d**) PCR-amplify the genomes of 40 randomly isolated plaques with primer pairs P6 targeting the ICP0 gene and the GFP gene for verification of double knock-in. (**e**) The genome of d0-GFP was extracted and analyzed by PCR, and images of fluorescent protein and DAPI-stained nuclei are shown.

The HSV-1 genome contains two unique sequences (UL and US), each of which is flanked by a pair of inverted repeat regions (TRL/IRL and IRS/TRS). There are two copies of each of genes on the terminal repeat long (TRL) regions, such as the ICP34.5, ICP0 and LAT genes. HSV-1 recombinants with ICP0 and ICP34.5 deletions were proven to possess enhanced oncolytic activity and immune boosting properties. Therefore, the substitution of these two genes has been widely used for the development of oncolytic viruses. Given the difficulty replacing two copies of the same gene on a single genome, the efficiency of this synchronous replacement is very low when using traditional recombination methods. However, the efficiency of replacing two copies of the same gene in the context of CRISPR/Cas9 technology has not been previously evaluated. To test the feasibility of a double replacement using CRISPR/Cas9-mediated editing of the HSV-1 genome, we employed the donor plasmid picp0EGFP and a gRNA-3 targeting the ICP0-coding region (Fig. 6a). The picp0EGFP plasmid was transfected into the 293T-gRNA-3 cells for 24 hours, and the cells were then infected with KOS viruses for another 24 hours before harvesting. Both ICP0 genes of HSV-1 were cleaved twice by the stably expressed Cas9/gRNA-3 complex, and the GFP gene was predicted to replace the ICP0 genes at both of their locations. First, we tested the efficiency of CRISPR/Cas9-mediated HDR of the ICP0 gene in the HSV-1 genome, one or two copies of the ICP0 gene could be replaced by the GFP gene, and the proportion of GFP^+^ cells was calculated to be ~1.8% after the reinfection of viruses at a MOI of below 0.5 (Fig. 6b). The efficiency of CRISPR/Cas9-mediated HDR of the ICP0 gene was significantly increased to approximately 1% (Fig. 6c). Moreover, the efficiency of CRISPR/Cas9-mediated editing of the two copies of the ICP0 gene at different locations in the HSV-1 genome was further investigated. We designed two different primer pairs to amplify the GFP gene and ICP0 gene, separately. If a double HDR event occurred, the ICP0 gene could be totally knocked-out and replaced with the GFP gene, so we can only amplify the GFP gene rather than the ICP0 gene using the above-mentioned primer pairs. If a single HDR event occurred, we can both amplify the GFP gene and ICP0 gene. If rearrangements or non-HDR events occurred, one copy of the ICP0 gene should be amplified at least. In order to verify the double replacement events, we randomly selected 40 GFP^+^ plaques isolated from the sorting GFP^+^ cells for PCR analysis of both the GFP and ICP0 genes. Our data revealed that 34 of them were confirmed to contain the GFP gene but no ICP0 gene, which suggest two copies of the ICP0 genes were completely knocked-out and replaced with the GFP gene (GFP^+^ ICP0^−^) (Fig. 6d). In this study, the proportion of GFP^+^ ICP0^−^ viruses was calculated to be ~85% (34 out of 40), so the efficiency of CRISPR/Cas9-mediated HDR of the two copies of the ICP0 gene was approximately 0.85%, which has never been reported before. The GFP^+^ ICP0^−^ viruses were designated as d0-GFP. An individual plaque of d0-GFP recombinant viruses was further verified by PCR and observed by fluorescence microscopy (Fig. 6e). In addition, 6 of them were confirmed to contain both the GFP gene and ICP0 gene, which suggest only one copy of the ICP0 gene was replaced with GFP (GFP^+^ ICP0^+^) or rearrangements occurred (Fig. S6). For excluding the rearrangements or non-HDR events, the fragments amplified from GFP^+^ ICP0^+^ viruses using a primer pair flanking the homolog arms were subjected to sequencing. It is confirmed that the large size fragment contained the ICP0 gene and the small size fragment contained the GFP gene, which may exclude the rearrangements or non-HDR mediated knockin.

### Evaluating the off-target effect of CRISPR/Cas9-mediated HSV genome editing

**Figure 7:**
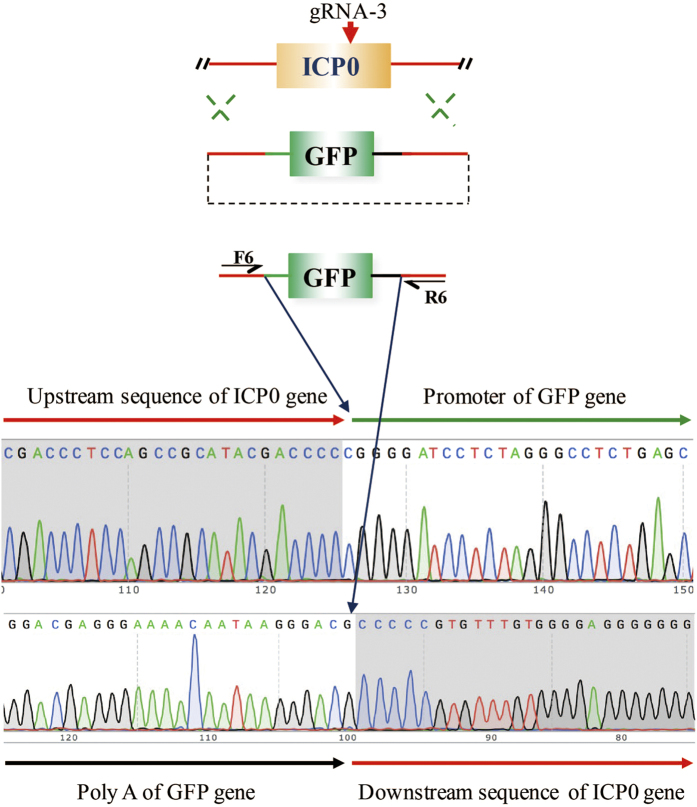
Intact sequence of the seam region between the homologous ends and the insert fragments.

Because of the off-target activity of Cas9, we aligned the target gRNA with the whole HSV-1 genome using a program that enables specific gRNA selection to minimize any potential off-target effects in the HSV-1 genome. Genome-wide gRNA libraries for the HSV-1 genome were designed using a previously described method^20^, and 19278 gRNAs in total were found for HSV-1 (Table S1). Based on previous reports that non-specific cleavage of CRISPR-Cas9 is sensitive to the positions and number of mismatches, this program employs an algorithm that calculates the sequence similarities between the input gRNA and HSV-1 gRNA libraries, and it will output a list of gRNA homologous sequences ranked from highest similarity score to lowest similarity score. The homologous sequence we considered for off-target evaluation contained ≤10 mismatches. We employed this program to select a specific gRNA for our target on the HSV-1 genome and launched an approach to evaluate the off-target activity of Cas9. This approach involved PCR-amplification of a region containing homologous sequences followed by deep sequencing. Among the three gRNAs we selected in this study, the smallest number of mismatched base pairs was seven (Table 1), possibly due to the use of program-mediated selection in advance. Using a gRNA-1-edited HSV-1 genome as the object, deep sequencing was performed on the highly homologous regions, and no off-target mutations were detected. Furthermore, we used another approach to evaluate the integrity of the seamless gene replacement. The d0-GFP was used as an object, and deep sequencing was performed on the upstream seam region and down stream seam region, which were PCR-amplified using the primer pair F6/R6. No unwanted mutations were detected in either seam region (Fig. 7), which suggested both gene copies on each single HSV-1 genome were seamlessly repaired by CRISPR/Cas9-mediated editing.

## Discussion

The CRISPR/Cas9 system has been shown to have greater feasibility and specificity than other techniques and greatly revolutionizes many fields of biomedical research^21^,^22^,^23^,^24^,^25^.

In this study we demonstrated the CRISPR/Cas9-mediated gene editing of the HSV-1 genome can be accomplished in human cells stably expressing CRISPR/Cas9. Under the guidance of gRNA, the selected PAM motif region on the HSV-1 genome was successfully edited, resulting in the deletion or insertion of random nucleotides. The editing was reasonably efficient, and no off-target cleavage was noted. Recombinant viruses with the desired GFP marker were isolated through single-cell sorting. Additionally, the NHEJ inhibitor SCR7 successfully increased the efficiency of CRISPR/Cas9-mediated gene replacement. This provided the first genetic evidence that two copies of the same gene in a single HSV-1 genome could be simultaneously efficiently edited through HDR-mediated gene replacement. ICP34.5 or ICP0 double- deleted HSV-1 recombinants have been proven to be good oncolytic viruses with enhanced oncolytic, immune-stimulating and anti-tumour properties^26^,^27^,^28^,^29^. Using our method, ICP0-null HSV-1 recombinant viruses with foreign genes could be created precisely and efficiently, which could be further developed as oncolytic viruses. Our work provides a new tool for precise and efficient genetic modification of HSV-1 and other DNA viruses. CRISPR/Cas9 technology might also facilitate the development of anti-cancer oncolytic viruses as well as antiviral agents and vaccines.

CRISPR/Cas9-mediated editing of the HSV-1 genome in human cells provides a new technology platform for genetic studies of HSV-1. Particularly, this approach will facilitate rapid study of the roles of individual HSV-1 genes in viral infection, replication and toxicity. BAC recombination technology used to be the most common method to modify the HSV-1 genome in *E. coli.*; however, several disadvantages associated with this method have been found in genetic studies of HSV-1^30^. First, genetic modifications of the HSV-1 genome are restricted to currently available HSV-1 BACs, which are not suitable for any HSV-1 strain. Second, the gene-editing pattern of HSV-1 genomes in prokaryotic cells is somehow different than that produced in eukaryotic cells, with unpredictable introductions of unknown epigenetic modifications and mutations. Third, the BAC technique requires the insertion of an additional selection marker into HSV-1 genome, which may affect genome integrity. Traditional transfection/infection of human cells is a good method for lossless gene editing, which largely depends on the occurrence rate of random recombination (lower than 0.05%). However, due to the low efficiency of HDR, the screening process for clones of interest when using this method is difficult, and labour-intensive work is required for verification. Thus, our work provides a proof-of-principle that gRNA-guided editing of the HSV-1 genome can improve the HDR frequency and the subsequently augment the production of HSV-1 recombinants of interest.

CRISPR/Cas9-mediated gene editing of adenovirus, Epstein-Barr virus (EBV) and Adeno-Associated virus (AAV) genomes has recently been reported. These studies have indicated the susceptibility of linear viral DNA to CRISPR/Cas9-mediated editing^18^,^31^,^32^. In previous work on HSV-1, which was focused on knocking-in genes, the homologous arms had the most proximity to the cleavage site, so the efficiency of HDR-mediated recombination was relatively high^17^,^33^. Besides that, our study also showed that CRISPR/Cas9 system can precisely and efficiently replace the genes in the HSV-1 genome followed by cell sorting and sequencing confirmation. In our method, the conditions and procedures were systematically investigated and optimized. Moreover, our study mainly focused on answering whether and how CRISPR/Cas9 method can precisely and efficiently make single or double gene replacements in the HSV-1 genome by HDR, which was more practical for constructing HSV-1 vectors. As a novel method, our results were obtained from cells stably expressing CRISPR/Cas9 that were infected with HSV-1. In these cells, both single-copy and double-copy genes could be knocked out by full replacement with desired genes with higher efficiency, possibly due to the relative high level and coverage of Cas9/gRNA components in the established cells during the short period of infection. In our system, the efficiency of gene replacement was improved by at least 10-fold, and DNA fragments of up to 4 kb were deleted or replaced. In addition, we described the rapid isolation of pure recombinant virus by reinfection coupled to single-cell sorting. This isolation was required for phenotypic characterization of the edited virus, such as GFP reporter. After final confirmation by whole genome sequencing, the correct clones were further selected as vectors for cancer therapy and vaccine development. Moreover, we reported the successful incorporation of the NHEJ inhibitor SCR7 into the CRISPR/Cas9-mediated editing of the HSV-1 genome. This significantly increased the HDR frequency in the presence of repair donor plasmids by counteracting NHEJ-directed repair, thereby facilitating subsequent homolog recombination between the viral genome and the repair template. In this regard, at least one desired virus could be isolated from every ten viruses.

Although the method developed for CRISPR/Cas9-mediated editing of the HSV-1 genome was highly successful, our results also raised new questions and suggested several new directions for further investigations. First, off-target cleavage remains a major concern in the application of CRISPR/Cas9 technology^34^. In our method, the off-target effect of CRISPR/Cas9 mediated genome editing of HSV-1 virus was unusually low, which was different from the results previously reported in editing other organisms, such as human cells. Here, we show that off-target effects of CRISPR/Cas9 can be reduced below the detection limits of deep sequencing by choosing unique target sequences in the HSV-1 genome. Meanwhile, it also highlights the importance of reducing the off-target effect of CRISPR/Cas9 system in gene editing. Recently, great advances have been achieved in reducing the off-target effect of nuclease-based genome editing, several novel nucleases had been developed with improved specificity, which would further improve our established method for targeted editing of DNA viruses. Although no off-target cleavage was detected by deep sequencing of highly homologous regions in our study, the nature and extent of the off-target effects potentially produced by CRISPR/Cas9-mediated editing of the HSV-1 genome warrants further investigation, including sequencing of the whole genome. Second, because of the high efficiency of CRISPR/Cas9-mediated editing of the HSV-1 genome, a library of gRNAs targeting to all potential sites on the HSV-1 genome could be designed to establish a panel of HSV-1 attenuated mutants, which could facilitate the development of vaccines against herpes viruses and oncolytic vectors^35^.

For the above reasons, CRISPR/Cas9 technology is a powerful and versatile tool for the targeted engineering of HSV-1 and other DNA viruses with large genomes.

## Methods

### Plasmids

The CRIPSR/Cas9 system used in this study was constructed by introducing synthesized oligo primers corresponding to the separate targets into a lentiCRISPR plasmid (Addgene, Cambridge, MA, USA). All guide sequences were selected by containing minimal mismatches to any mammalian genomic sequences or unrelated HSV-1 genomic sequences. The target sites and gRNA sequences used in this study are shown in Table 1.

For the construction of the donor plasmids, different strategies were used. To generate the pTUL37/38 plasmid, the UL37 region and the UL38 region were separately amplified with the primer pairs UL37F/R and UL38F/R and hen sequentially cloned into multiple sites in a pMD18-T vector using the Gibson Assemble method. To generate the pTUL37/38GFP, pTUL37/38mCherry and pTUL37/38lacZ plasmids, fragments containing CMV-EGFP-BGH, CMV-mCherry-BGH and CMV-lacZ-BGH were amplified from pcDNA3.1-EGFP, pcDNA3.1-mCherry and pcDNA3.1-lacZ, respectively, using the primer pair CMVF/BGHR. These fragments were then inserted into the linker site of pTUL37/38 between the UL37 and UL38 regions using the restriction enzyme PacI. To generate the picp0 plasmid, the 5′ and 3′ regions flanking the genomic regions of ICP0 were amplified with the primer pair RL2RAF/RL2LAR and sequentially cloned into multiple sites in a pMD18-T vector. To generate the picp0EGFP plasmid, the ICP0 region of picp0 was replaced with DNA fragments encoding EGFP using the restriction enzymes NcoI and SalI. These fragments were amplified from pcDNA3.1-EGFP using the primer pair CMVF1/BGHR2.

For BstZ17I enzymatic analysis, the fragments were amplified using the primer pair F1/R1. For verification of recombinant viruses, five pairs of verification primers p1 (F2 + R2), p2 (F3 + R3), p3 (F4 + R4), p4 (F5 + R5), p5 (F6 + R6) and p6 (F7 + R7), were used to separately PCR-amplify the GFP gene (720 bp for p1), the UL37/UL38 homologous replacement region (2105 bp for p2), the LacZ gene (3049 bp for p3), the ICP0 gene (2560 bp for p4; 1147 bp for p6) and the ICP0 homologous replacement region (860 bp for p5). All primers used in this study are listed in Table S2.

### Cell culture and transfection

HEK293T and U-2 OS cells were grown in DMEM supplemented with 10% heat-inactivated FBS and antibiotics at 37 °C and 5% CO2. For lentivirus production, lentiCRISPR plasmids were co-transfected into HEK293T cells with the packaging plasmids pMD2.G and psPAX2 using PEI reagent. After a 48-h transfection, supernatants were collected and clarified by passing through 0.45-μm filters, and the virus was stored at −80 °C. For lentivirus transduction, HEK293T cells were infected with prepared lentivirus and cultured in DMEM medium containing 1 μg/mL puromycin for 3 days. Stably transfected 293T-gRNA cells were then expanded and maintained in DMEM medium containing 0.5 μg/mL puromycin.

### CRISPR/Cas9-mediated genome editing

293T-gRNA1, 293T-gRNA2 and 293T-gRNA3 cells were transiently transfected with donor plasmids or mock plasmids using Lipofectamine 2000 (Invitrogen). After a 24 hours’ transfection, the cells were infected with HSV-1 virus at different MOIs for 24 h. The infected cells were harvested at the indicated time points and subjected to two rounds of freeze and thaw cycles. The titers of the amplified viruses were determined on U-2 OS cells using a classic plaque assay. In brief, a monolayer of U-2 OS cells at a density of 2 × 10^6^ cells per 6 cm dish was infected with serially diluted virus in a volume of 0.5 mL for 1.25 h. After viral entry, the cells were overlaid with 2% methylcellulose medium and incubated at 37 °C in 5% CO_2_ for 3 days. Then, the dishes were stained with neutral red for overnight, and the plaques were counted manually using a white-light transilluminator. Viral titers (PFU/mL) were calculated using the equation plaque numbers × dilution fold × 2. For screening LacZ^+^ plaques, the dishes were stained with a mixture of neutral red solution and X-Gal (5-bromo-4-chloro-3-indolyl-β-D-galacto-pyranoside) until blue plaques appeared. For screening GFP^+^ and mCherry^+^ plaques, plaques were either examined by fluorescence microscopy or infected GFP^+^ and mCherry^+^ cells were sorted using a BD FACSAria II cell sorter. The titers of GFP^+^ and mCherry^+^ viruses were calculated using the equation fluorescence plaque numbers × dilution fold × 2 or the equation the percentage of green cells × 2 × 10^6^ × viral titer/input virus.

### PCR analysis and sequencing analysis

HSV-1 genomic DNA was extracted by using the QIAamp DNA Blood MiniKit (Qiagen). The genomic region surrounding the cleavage sites of each gene was amplified using KOD-plus high-fidelity DNA polymerase (Toyobo) with the primers listed in Table S2. To analyse restriction fragment length polymorphisms, PCR products were digested by BstZ17I and visualized on a 3% agarose gel. Quantification was based on relative band intensities by Image J software. All experiments were performed in triplicates. The identities of PCR products were confirmed by direct DNA sequencing.

### NHEJ inhibitor treatment and HDR analysis

Cells stably expressing gRNA-2 were transfected with linearized pTUL37/38lacZ and treated with SCR7 (Excess Bioscience) at 10 μM or vehicle (DMSO). After 24 h, the cells were infected with the indicated HSV-GFP viruses for an additional 24 h and then collected and processed for titration. To analyse the frequency of NHEJ-directed repair, the percentage of GFP^−^ LacZ^−^ viruses was counted. To analyse the frequency of HDR-mediated repair, the percentage of GFP^−^LacZ^+^ viruses was counted. The titers of LacZ^+^ viruses and GFP^+^ viruses were calculated using the above-mentioned methods for screening LacZ and GFP plaques.

## Additional Information

**How to cite this article**: Lin, C. *et al*. Increasing the Efficiency of CRISPR/Cas9-mediated Precise Genome Editing of HSV-1 Virus in Human Cells. *Sci. Rep.* **6**, 34531; doi: 10.1038/srep34531 (2016).

## Supplementary Material

Supplementary Information

Supplementary Information

## Figures and Tables

**Table 1:** Numbers of mutations detected at the potential off-target sites in the HSV-1 genome.

gRNA ID	Targets	gRNA Coding Sequence	No. of mismatches	Off-target sites	Mutations
				Location: bp	
gRNA-1	UL37-UL38	GCCCTTTAAACGTGTGTATACGG	0	KOS: 84287-84306	
		GCCCTTTAAAGGCGGGCGGCGGG	7	KOS: 374-396	None detected
		CGGCGAAGAACGTGTGTGCGGGG	10	KOS: 79986-80008	None detected
gRNA-2	EGFP	GCTGAAGCACTGCACGCCGTAGG	0	GFP: 219-200	
		GGGGGCCTGTTGCACGCCGAAGG	9	KOS: 76596-76574	None detected
gRNA-3	RL2 (ICP0)	GCTCCATGGGGGTCGTATGCGG	0	KOS: 2241-2223; 124000-124018	
		None	≤10		None detected
