# Supplementary material for: Increasing the Efficiency of CRISPR/Cas9-mediated Precise Genome Editing of HSV-1 Virus in Human Cells
Source: Sci Rep. 2016 Oct 7;6:34531. doi: 10.1038/srep34531 (PMC5054376; doi:10.1038/srep34531)
Supplement: Supplementary Information [file srep34531-s1.docx]

**Increasing the Efficiency of CRISPR/Cas9-mediated Precise Genome Editing of HSV-1 Virus in Human Cells**

**Chaolong Lin**1**, Huanhuan Li**2**, Mengru Hao**2**, Dan Xiong**2**, Yong Luo**1**, Chenghao Huang**1,***, Quan Yuan**1**, Jun Zhang**1**, and Ningshao Xia**1,2,*

1State Key Laboratory of Molecular Vaccinology and Molecular Diagnostics, National Institute of Diagnostics and Vaccine Development in Infectious Diseases, School of Public Health, Xiamen University, Xiamen, 361102, China

2School of Life Sciences, Xiamen University, Xiamen, 361102, China

* huangchenghao@xmu.edu.cn or nsxia@xmu.edu.cn

| **Table S2. Primers used in this study** | |
| --- | --- |
| **Name** | **Sequence** |
|  |  |
| UL37F | TGCAGGTCGACGATTCGACGAACTCAGGAACGGCA |
| UL37R | GTTTAAACTTAATTAAGAAAGTCCGCAACCCACGCCGT |
| UL38F | TTAATTAAGTTTAAACGGCGGGGCGGCCCAAATGGCCCTTTAAACGTGTGTATA |
| UL38R | GGTACCCGGGGATCCTCTAGATCACGCGCAGGCC |
| CMVF | GCGGACTTTCTTAATTAATATTAATAGTAATCAATTACG |
| BGHR | GGCCGCCCCGCCTTAATTAAGGATCCAGACATGATAAGATA |
| RL2RAF | CATGATTACGCCAAGCTTGAGCTCCGCACCAAGCCGCTCTC |
| RL2LAR | ACGACGGCCAGTGAATTCGTTAACACCAGAGCCTGCCCAA |
| CMVF1 | CGCATACGACCCCCATGGTCGCGAATGGTGAGCAAGGGCGAGGA |
| BGHR2 | CCCTCGTCCCGGGTCGACTTACTTGTACAGCTCGTCCATGCCGA |
| gRNA1F | CACCGCCCTTTAAACGTGTGTATA |
| gRNA1R | AAACTATACACACGTTTAAAGGGC |
| gRNA2F | CACCGCTGAAGCACTGCACGCCGT |
| gRNA2R | AAACACGGCGTGCAGTGCTTCAGC |
| gRNA3F | CACCGGCTCCATGGGGGTCGTATG |
| gRNA3R | AAACCATACGACCCCCATGGAGCC |
| F1 | CTAGGCTCGCCAATAGGCGCT |
| R1 | GCGGGTAGCGGATTGGTCTTCATTG |
| F2 | ATGGTGAGCAAGGGCGAGGAGCTGTT |
| R2 | TTACTTGTACAGCTCGTCCATGCCGAG |
| F3 | CACAGGTGTTCCAGCAGCG |
| R3 | GGATCTTGTCCAGGGCCTGTTC |
| F4 | GGGCCAGATATACGCGTTGAC |
| R4 | AGTGGATCCAGACATGATAAG |
| F5 | ATGGAACCACGTCCAGGAGCAAGTA |
| R5 | TTATTGTTTTCCCTCGTCACGGGTCGAC |
| F6 | GACAGTCTGGTCGCATTTGCACCTC |
| R6 | CAACACAAAAGACCCGCTGGTGTGTG |
| F7 | GACGTGTGCGCCGTGTGCACGGATGA |
| R7 | ACTCTGTTCTTGGTTCGCGGCCTGAGCCA |

**Figure S1. BstZ17I enzymatic analysis of fragments amplified from cells harvested at different time points.**

**Figure S2. BstZ17I enzymatic analysis of fragments amplified from cells infected with different MOIs.**

**Figure S3. Editing of the HSV-GFP genome in 293T cells stably expressing CRISPR/Cas9.**

**Figure S4. Images of cells infected with the mutated parental HSV-GFP viruses after CRISPR/Cas9 editing at MOI of 1 PFU/cell were shown.**

Image under normal light Image of fluorescence

**
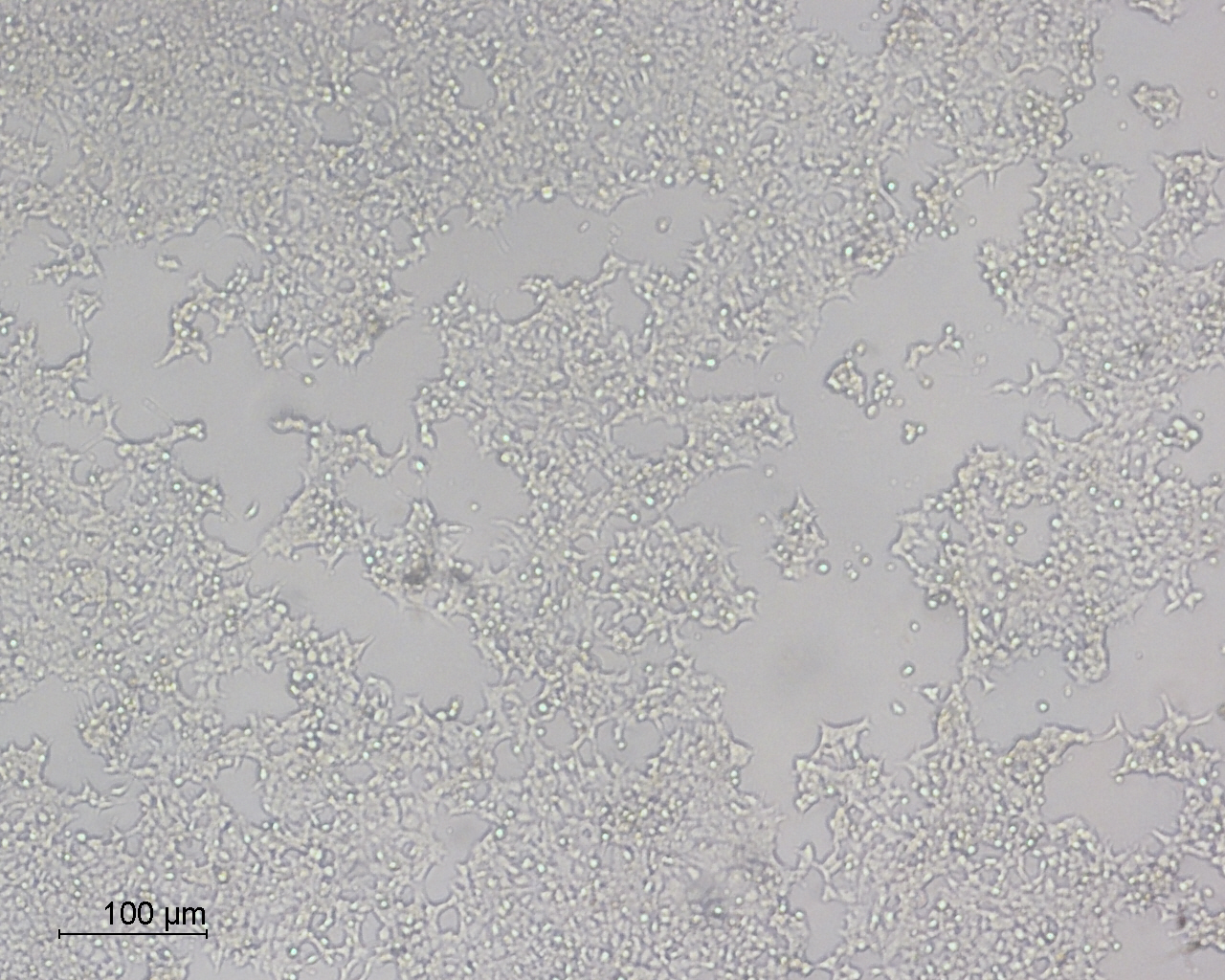

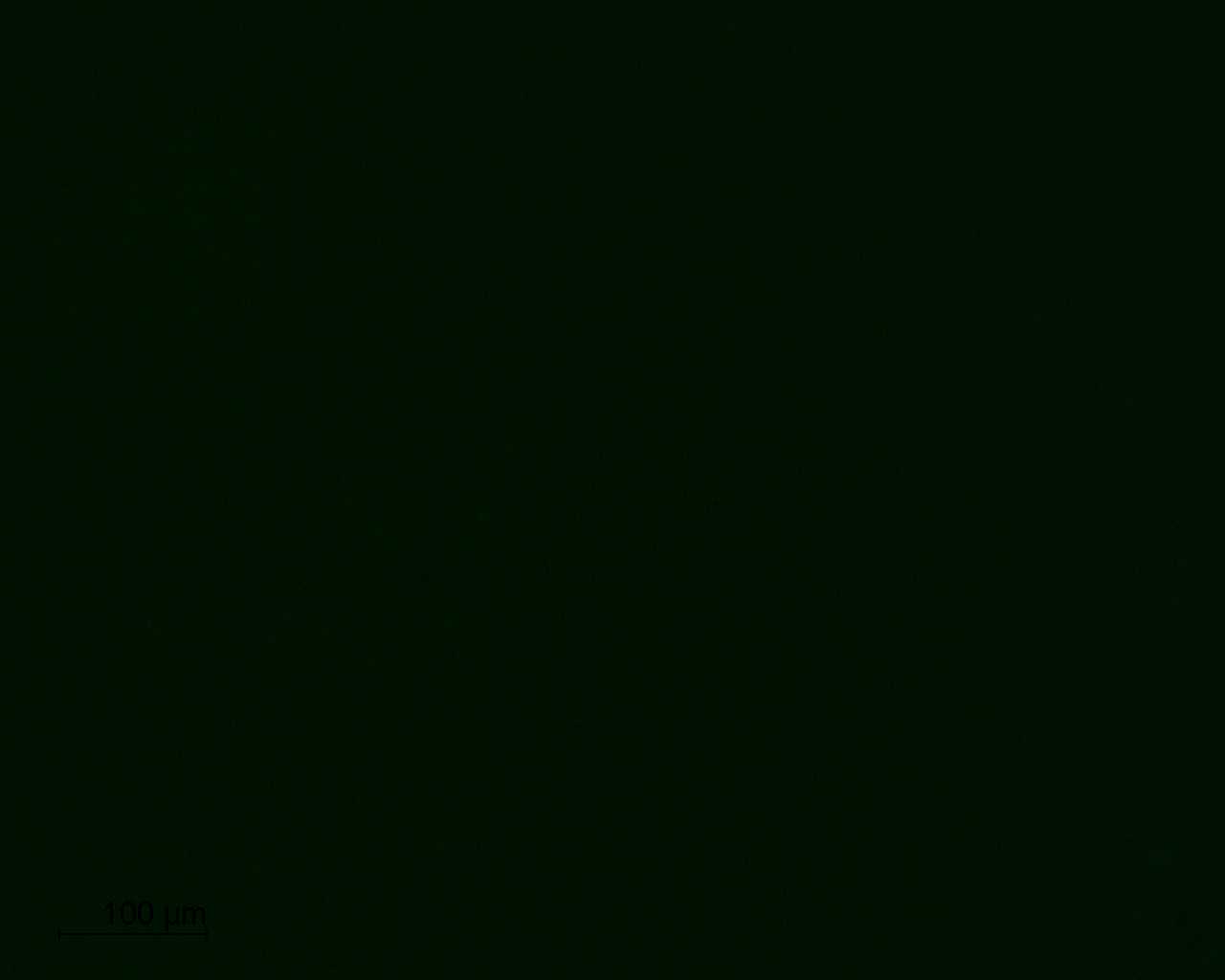
**

**Figure S5. Strategy for separating the HSV-mutGFP viruses (GFP^-^) from the mixed pool of edited HSV-GFP viruses.**

**Figure S6. The fragments amplified from the edited HSV-GFP viruses using a primer pair flanking the homolog arm. The large size fragment contained the ICP0 gene and the small size fragment contained the GFP gene.**

**7500**

**5000**

**2500**

**1000**

**750**


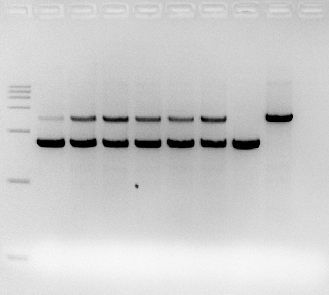


**Marker**

**14**

**16**

**24**

**30**

**34**

**39**

**KOS**

**CTL**

**Repair**

**donor**
